# Bias in exposure-outcome associations when using left truncated birth datasets.

**DOI:** 10.23889/ijpds.v7i3.1789

**Published:** 2022-08-25

**Authors:** Jennifer Dunne, Gizachew Tessema, Gavin Pereira

**Affiliations:** 1 Curtin University

## Objectives

Restriction to the analysis of births that survive past a specified gestational age (typically 20 weeks gestation) can lead to biased exposure-outcome associations. The objective is to estimate the influence of bias resulting from using a left truncated dataset to ascertain exposure-outcome associations in perinatal studies.

## Approach

We simulated the magnitude of bias under a collider-stratification mechanism for the association between the exposure of advancing maternal age (≥ 35 years) and the outcome of stillbirth. This bias occurs when the cause of restriction (early pregnancy loss) is influenced by both the exposure and unmeasured factors that also affect the outcome. Simulation parameters were based on an original birth cohort from Western Australia and a range of plausible values for the prevalence of early pregnancy loss (< 20 gestational weeks), an unmeasured factor U and the odds ratios for the selection effects. Selection effects included the effects of maternal age on early pregnancy loss, U on early pregnancy loss, and U on stillbirth. We then compared the simulated scenarios with the results from the original cohort in which bias was unadjusted.

## Results

We found the overall magnitude of bias to be minimal in the association between advancing maternal age and stillbirth. The findings indicate that the stronger the effect of the unmeasured U on early pregnancy loss and stillbirth, the greater the departure from the null. When we compared the simulated model with the results of the original cohort, we found evidence of marginal downward bias which was most prominent for women aged 40+ years.

## Conclusion

Our simulations demonstrated a marginal downward bias in the association between advancing maternal age and stillbirth. We recommend that future studies should quantify the extent of such bias when using left truncated birth datasets to determine exposure-outcome associations.

